# Disilver(I) trinickel(II) hydrogenphos­phate bis­(phosphate), Ag_2_Ni_3_(HPO_4_)(PO_4_)_2_

**DOI:** 10.1107/S1600536811021167

**Published:** 2011-06-11

**Authors:** Abderrazzak Assani, Lahcen El Ammari, Mohammed Zriouil, Mohamed Saadi

**Affiliations:** a Laboratoire de Chimie du Solide Appliquée, Faculté des Sciences, Université Mohammed V-Agdal, Avenue Ibn Battouta, BP 1014, Rabat, Morocco

## Abstract

The title compound, Ag_2_Ni_3_(HPO_4_)(PO_4_)_2_, has been synthesized by the hydro­thermal method. Its structure is formed by two types of chains running along the *b* axis. The first chain results from a linear and continuous succession of NiO_6_ octa­hedra linked to PO_4_ tetra­hedra by a common vertex. The second chain is built up from two adjacent edge-sharing octa­hedra (dimers) whose ends are linked to two PO_4_ tetra­hedra by a common edge. Those two types of chains are linked together by the phosphate groups to form polyhedral sheets parallel to the (001) plane. The three-dimensional framework delimits two types of hexa­gonal tunnels parallel to the *a*-axis direction, at (*x*, 1/2, 0) and (*x*, 0, 1/2), where the Ag atoms are located. Each silver cation is surrounded by eight O atoms. The same Ag^+^ coordination is found in other phosphates with the alluaudite structure, for example, AgMn_3_(PO_4_)(HPO_4_)_2_. Moreover, O—H⋯O hydrogen bonds link three PO_4_ tetra­hedra so as to build a three-dimensional network.

## Related literature

For related applications, see: Viter & Nagornyi (2009 ▶); Gao & Gao (2005 ▶); Clearfield (1988 ▶); Trad *et al.* (2010 ▶). For compounds with the same structure, see: Assani *et al.* (2010 ▶, 2011 ▶); Leroux *et al.* (1995 ▶); Ben Smail & Jouini (2002 ▶).

## Experimental

### 

#### Crystal data

Ag_2_Ni_3_(HPO_4_)(PO_4_)_2_
*M* *_r_* = 677.79
Orthorhombic, 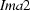

*a* = 12.9233 (3) Å
*b* = 6.5678 (2) Å
*c* = 10.6629 (3) Å
*V* = 905.04 (4) Å^3^
*Z* = 4
Mo *K*α radiation
μ = 10.98 mm^−1^
*T* = 296 K
0.25 × 0.13 × 0.08 mm

#### Data collection

Bruker X8 APEXII CCD area-detector diffractometer
Absorption correction: multi-scan (*MULABS*; Blessing, 1995 ▶) *T*_min_ = 0.382, *T*_max_ = 0.471
3762 measured reflections
1125 independent reflections
1103 reflections with *I* > 2σ(*I*)
*R*_int_ = 0.017

#### Refinement

*R*[*F* ^2^ > 2σ(*F* ^2^)] = 0.024
*wR*(*F* ^2^) = 0.060
*S* = 1.06
1125 reflections
99 parameters
1 restraint
H-atom parameters constrained
Δρ_max_ = 1.81 e Å^−3^
Δρ_min_ = −1.12 e Å^−3^
Absolute structure: Flack (1983 ▶), 467 Friedel pairs
Flack parameter: 0.55 (3)

### 

Data collection: *APEX2* (Bruker, 2005 ▶); cell refinement: *SAINT*; data reduction: *SAINT*; program(s) used to solve structure: *SHELXS97* (Sheldrick, 2008 ▶); program(s) used to refine structure: *SHELXL97* (Sheldrick, 2008 ▶); molecular graphics: *ORTEP-3 for Windows* (Farrugia, 1997 ▶) and *DIAMOND* (Brandenburg, 2006 ▶); software used to prepare material for publication: *WinGX* (Farrugia, 1999 ▶).

## Supplementary Material

Crystal structure: contains datablock(s) I, global. DOI: 10.1107/S1600536811021167/ru2005sup1.cif

Structure factors: contains datablock(s) I. DOI: 10.1107/S1600536811021167/ru2005Isup2.hkl

Additional supplementary materials:  crystallographic information; 3D view; checkCIF report

## Figures and Tables

**Table 1:** Hydrogen-bond geometry (Å, °)

*D*—H⋯*A*	*D*—H	H⋯*A*	*D*⋯*A*	*D*—H⋯*A*
O7—H7⋯O6^i^	0.86	2.06	2.847 (6)	151

Symmetry code: (i) 
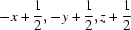
.

**Fig. 1.**
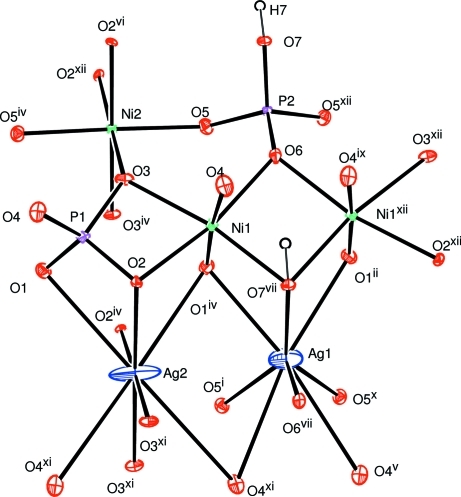
Partial plot of Ag2Ni3(HPO4)(PO4)2 crystal structure. Displacement ellipsoids are drawn at the 50% probability level. Symmetry codes: (i) -x, -y + 1, z; (ii) x + 1/2, -y + 1, z; (iii) x, -y + 3/2, z - 1/2; (iv) -x + 1/2, -y + 3/2, z - 1/2; (v) -x + 1/2, -y + 1/2, z - 1/2; (vi) -x, y + 1/2, z - 1/2; (vii) x + 1/2, y + 1/2, z - 1/2; (viii) x, -y + 1/2, z - 1/2; (ix) -x, -y, z; (x) -x, y + 1/2, z + 1/2; (xi) x, -y + 1/2, z + 1/2; (xii) -x + 1/2, y, z.

**Fig. 2.**
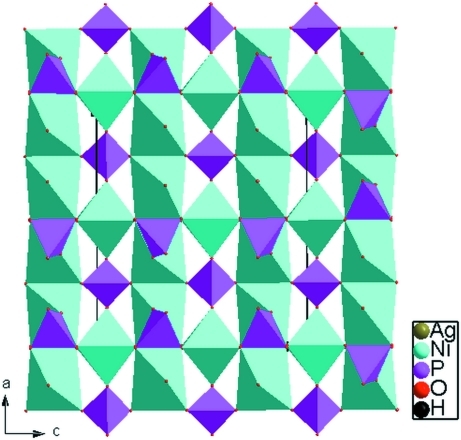
View along the b axis of polyhedral sheets parallel to the (0 0 1) plane.

**Fig. 3.**
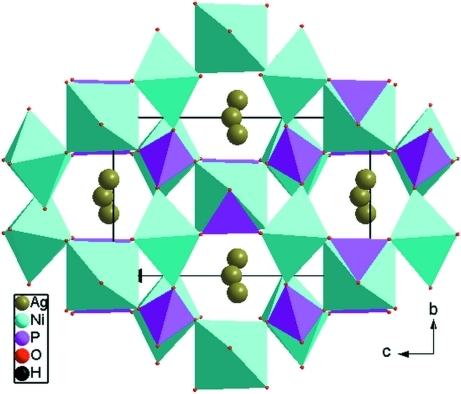
A three-dimensional polyhedral view of the crystal structure of the Ag2Ni3(HPO4)(PO4)2, showing tunnels running along the a direction, at x, 1/2, 0 and x, 0, 1/2.

## supplementary crystallographic information

### Comment

As the improvement has arisen in the synthesis of a variety of interesting porous materials and open-framework structures, extensive studies are devoted to the metal phosphates which exhibit a rich structural diversity and have been widely studied as catalysts (Viter & Nagornyi, 2009; Gao & Gao, 2005), ion-exchangers (Clearfield, 1988) and as positive electrode in the lithium and sodium batteries (Trad *et al.* (2010)).

Within this family of compounds, the resulting anionic frameworks, generally constructed from the alternation of PO_4_ tetrahedra connected to metal cations in different coordinate geometry MO_n_ (with n=4, 5 and 6), generate pores and channels offering suitable environment to accommodate different other cations. In our search for new phosphates with microporous framework, our most attention has been paid to the hydrothermal investigation of the A_2_O—MO—P_2_O_5_ systems, with A = monovalent cations and *M* = divalent cations. Accordingly, we have succeed, for instance, to isolate new form of silver zinc phosphate (γ-AgZnPO_4_) related to the ABW zeolite structure (Assani *et al.* 2010) while the silver magnesium phosphate, namely AgMg_3_(PO_4_)(HPO_4_)_2_, represent a new member of the well known alluaudite-like structure family (Assani *et al.* 2011). The present paper aims to develop the hydrothermal synthesis and the structural characterization of a new silver nickel phosphate, namely, Ag_2_Ni_3_(HPO_4_)(PO_4_)_2_.

The structure of this compound is formed by two types of chains running along the *b* axis. The first chain (Ni2P2HO_9_)∞ is built up from Ni2 and P2 atoms in special Wyckoff position 4 b (*m*) of the space group Ima2. This chain results from linear and continuous succession of octahedron (Ni2O_6_) and P2O_3_OH tetrahedron which share a vertex. The second chain (Ni_2_P_2_O_14_)n is built up from two adjacent edge sharing octahedra ((Ni1)_2_O_10_ dimmers) whose ends are linked to two P1O_4_ tetrahedra by a common edge (Fig.1). Those chains are linked together by the phosphate groups to form polyhedral sheets parallel to the (0 0 1) plane as shown in Fig.2.

The three dimensional framework delimits two types of hexagonal tunnels running along the a direction, at *x* 1/2 0 and *x* 0 1/2 (Fig.3). The Ag2 atom is located at centre of tunnels, this explains the high value of its anisotropic displacement U_11_, whereas Ag1 is slightly shifted from this center (Wyckoff positions: Ag2 at 2a: 0, 0, *z* and Ag1 at 2 b: 1/4, *y*, *z*). However, each Ag^+^ ion is surrounded by 8 O atoms with different Ag–O distances. Indeed, the first coordination environment of Ag2^+^ is almost square planar with four short Ag2—O distances between 2.373 (4) and 2.421 (4) Å and the other four larger distances are in the range of 2.869 (4) to 3.133 (4) Å. A similar coordination surrounding Ag1^+^ is observed with Ag1—O bond lengths in the range of 2.537 (4)–2.616 (4) Å and the longest bonds are situated between 2.661 (4) and 2.963 (4) Å. The same coordination for this cation is found in other phosphate with alluaudite structure like AgMn_3_(PO_4_)(HPO_4_)_2_ (Leroux *et al.* (1995)) and AgNi_3_(PO_4_)(HPO_4_)_2_ (Ben Smail & Jouini (2002)).

Moreover, O—H···O hydrogen bondings link two adjacent P2O_4_ tetrahedra *via* a strong hydrogen bond O7–H7···O6 to two P1O_4_ tetrahedra through weak bonds O7–H7···O4 in the way to build an infinite three-dimensional network as shown in Table 1.

### Experimental

By means of hydrothermal synthesis, we have isolate a new silver nickel phosphate from the reaction mixture of silver nitrate (AgNO_3_; 0.1699 g), metallic nickel (Ni; 0.0881 g), 85%wt phosphoric acid (H_3_PO_4_; 0,10 ml) and water (12 ml). The hydrothermal treatment was conducted in a 23 ml Teflon-lined autoclave under autogeneous pressure at 468 K for two days. After being filtered off, washed with deionized water and air dried, the reaction product consists of a monophasic green powder and some green parallelepipedic crystals corresponding to the title compound.

### Refinement

The structure is solved by direct method technique and refined by full-matrix least-squares using *SHELXS97* and *SHELXL97* program packages. The structure refinement in the centrosymmetric space group was unsuccessful. Infact the crystal is a racemic twinned with a refined ratio of 0.479 (26), which explains the ambiguity in the Flack parameter. The space group is not centro symmetric and the polar axis restraint is generated automatically by *SHELXL* program. Friedel opposites reflections are not merged. The O-bound H atom is initially located unambiguously in a difference map and refined with O—H distance restraints of 0.86 (1). In a the last cycle ther is refined in the riding model approximation with *U*_iso_(H) set to 1.2*U*_eq_(O). The highest and deepest hole residual peak in the final difference Fourier map are located at 0.72 Å and 0.62 Å, from Ag1.

### Crystal data

**Table:**

Ag_2_Ni_3_(HPO_4_)(PO_4_)_2_	*F*(000) = 1280
*M**_r_* = 677.79	*D*_x_ = 4.974 Mg m^−^^3^
Orthorhombic, *I**m**a*2	Mo *K*α radiation, λ = 0.71073 Å
Hall symbol: I 2 -2a	Cell parameters from 1125 reflections
*a* = 12.9233 (3) Å	θ = 3.2–29.0°
*b* = 6.5678 (2) Å	µ = 10.98 mm^−^^1^
*c* = 10.6629 (3) Å	*T* = 296 K
*V* = 905.04 (4) Å^3^	Prism, green
*Z* = 4	0.25 × 0.13 × 0.08 mm

### Data collection

**Table:**

Bruker X8 APEXII CCD area-detector diffractometer	1125 independent reflections
Radiation source: fine-focus sealed tube	1103 reflections with *I* > 2σ(*I*)
graphite	*R*_int_ = 0.017
φ and ω scans	θ_max_ = 29.0°, θ_min_ = 3.2°
Absorption correction: multi-scan (*MULABS*; Blessing, 1995)	*h* = −13→14
*T*_min_ = 0.382, *T*_max_ = 0.471	*k* = −16→17
3762 measured reflections	*l* = −8→8

### Refinement

**Table:**

Refinement on *F*^2^	Secondary atom site location: difference Fourier map
Least-squares matrix: full	Hydrogen site location: inferred from neighbouring sites
*R*[*F*^2^ > 2σ(*F*^2^)] = 0.024	H-atom parameters constrained
*wR*(*F*^2^) = 0.060	*w* = 1/[σ^2^(*F*_o_^2^) + (0.0356*P*)^2^ + 1.7344*P*] where *P* = (*F*_o_^2^ + 2*F*_c_^2^)/3
*S* = 1.06	(Δ/σ)_max_ < 0.001
1125 reflections	Δρ_max_ = 1.81 e Å^−^^3^
99 parameters	Δρ_min_ = −1.12 e Å^−^^3^
1 restraint	Absolute structure: Flack (1983), 467 Friedel pairs
Primary atom site location: structure-invariant direct methods	Flack parameter: 0.55 (3)

### Special details

**Table:**

Geometry. All e.s.d.'s (except the e.s.d. in the dihedral angle between two l.s. planes) are estimated using the full covariance matrix. The cell e.s.d.'s are taken into account individually in the estimation of e.s.d.'s in distances, angles and torsion angles; correlations between e.s.d.'s in cell parameters are only used when they are defined by crystal symmetry. An approximate (isotropic) treatment of cell e.s.d.'s is used for estimating e.s.d.'s involving l.s. planes.
Refinement. Refinement of *F*^2^ against ALL reflections. The weighted *R*-factor *wR* and goodness of fit *S* are based on *F*^2^, conventional *R*-factors *R* are based on *F*, with *F* set to zero for negative *F*^2^. The threshold expression of *F*^2^ > σ(*F*^2^) is used only for calculating *R*-factors(gt) *etc*. and is not relevant to the choice of reflections for refinement. *R*-factors based on *F*^2^ are statistically about twice as large as those based on *F*, and *R*- factors based on ALL data will be even larger.

### Fractional atomic coordinates and isotropic or equivalent isotropic displacement parameters (Å^2^)

**Table:**

	*x*	*y*	*z*	*U*_iso_*/*U*_eq_	
Ag1	0.2500	0.60793 (8)	−0.01513 (7)	0.02914 (18)	
Ag2	0.0000	0.5000	−0.03769 (5)	0.0443 (2)	
Ni1	0.13623 (4)	0.24801 (10)	0.20871 (7)	0.00699 (13)	
Ni2	0.0000	0.5000	0.45735 (7)	0.00462 (15)	
P1	−0.07279 (7)	0.25722 (19)	0.20677 (13)	0.00587 (19)	
P2	0.2500	0.4102 (2)	0.45653 (15)	0.0042 (3)	
O1	−0.1343 (3)	0.4456 (5)	0.1739 (3)	0.0091 (7)	
O2	0.0044 (3)	0.2070 (6)	0.1000 (3)	0.0056 (6)	
O3	0.0036 (3)	0.2785 (5)	0.3204 (3)	0.0072 (8)	
O4	−0.1494 (3)	0.0786 (5)	0.2360 (3)	0.0096 (9)	
O5	0.1543 (2)	0.5443 (4)	0.4552 (3)	0.0085 (5)	
O6	0.2500	0.2617 (8)	0.3420 (5)	0.0090 (12)	
O7	0.2500	0.2692 (7)	0.5756 (5)	0.0064 (12)	
H7	0.2500	0.3065	0.6529	0.008*	

### Atomic displacement parameters (Å^2^)

**Table:**

	*U*^11^	*U*^22^	*U*^33^	*U*^12^	*U*^13^	*U*^23^
Ag1	0.0491 (4)	0.0158 (3)	0.0225 (3)	0.000	0.000	0.0048 (3)
Ag2	0.1145 (7)	0.0083 (2)	0.0101 (3)	−0.0020 (3)	0.000	0.000
Ni1	0.0058 (2)	0.0092 (3)	0.0060 (3)	0.0005 (2)	0.0005 (3)	−0.00135 (19)
Ni2	0.0052 (3)	0.0049 (3)	0.0037 (3)	0.0006 (2)	0.000	0.000
P1	0.0064 (4)	0.0064 (5)	0.0047 (4)	−0.0001 (5)	0.0008 (6)	0.0004 (4)
P2	0.0043 (6)	0.0054 (6)	0.0030 (7)	0.000	0.000	−0.0012 (6)
O1	0.0086 (16)	0.0100 (18)	0.0088 (15)	0.0003 (14)	−0.0017 (10)	−0.0010 (12)
O2	0.0044 (18)	0.0088 (15)	0.0035 (15)	−0.0002 (15)	−0.0010 (12)	−0.0029 (13)
O3	0.012 (2)	0.0044 (17)	0.0049 (15)	0.0009 (15)	−0.0005 (12)	−0.0010 (12)
O4	0.010 (2)	0.0023 (18)	0.016 (2)	−0.0011 (13)	0.0015 (11)	0.0017 (11)
O5	0.0077 (12)	0.0082 (12)	0.0096 (15)	0.0014 (10)	−0.0008 (13)	−0.0003 (12)
O6	0.005 (3)	0.013 (3)	0.009 (2)	0.000	0.000	−0.0007 (16)
O7	0.007 (3)	0.007 (3)	0.005 (2)	0.000	0.000	0.0023 (16)

### Geometric parameters (Å, °)

**Table:**

Ag1—O1^i^	2.534 (3)	Ni2—O2^xi^	2.041 (3)
Ag1—O1^ii^	2.535 (3)	Ni2—O3^i^	2.062 (4)
Ag1—O5^iii^	2.617 (3)	Ni2—O3	2.062 (4)
Ag1—O5^iv^	2.617 (3)	P1—O1	1.511 (4)
Ag1—O7^v^	2.659 (5)	P1—O2	1.549 (4)
Ag1—O6^v^	2.866 (5)	P1—O4	1.566 (4)
Ag1—O4^vi^	2.962 (3)	P1—O3	1.569 (4)
Ag1—O4^vii^	2.962 (3)	P2—O5	1.518 (3)
Ag1—Ag2	3.31641 (16)	P2—O5^xii^	1.518 (3)
Ag2—O3^vi^	2.375 (4)	P2—O6	1.563 (6)
Ag2—O3^viii^	2.375 (4)	P2—O7	1.572 (5)
Ag2—O2	2.421 (4)	O1—Ni1^i^	2.046 (4)
Ag2—O2^i^	2.421 (4)	O1—O4	2.507 (5)
Ag2—O1	2.869 (4)	O1—Ag1^i^	2.534 (3)
Ag2—O1^i^	2.869 (4)	O2—Ni2^xiii^	2.041 (3)
Ag2—O4^viii^	3.133 (4)	O3—Ag2^xiv^	2.375 (4)
Ag2—O4^vi^	3.133 (4)	O4—Ni1^ix^	2.172 (4)
Ni1—O1^i^	2.046 (4)	O4—Ag1^xv^	2.962 (3)
Ni1—O7^v^	2.047 (3)	O4—Ag2^xiv^	3.133 (4)
Ni1—O6	2.047 (4)	O5—Ag1^xvi^	2.617 (3)
Ni1—O2	2.079 (4)	O6—Ni1^xii^	2.047 (4)
Ni1—O3	2.097 (4)	O6—Ag1^xvii^	2.866 (5)
Ni1—O4^ix^	2.172 (4)	O7—Ni1^xi^	2.047 (3)
Ni2—O5^i^	2.015 (3)	O7—Ni1^xvii^	2.047 (3)
Ni2—O5	2.015 (3)	O7—Ag1^xvii^	2.659 (5)
Ni2—O2^x^	2.041 (3)	O7—H7	0.8600
			
O1^i^—Ag1—O1^ii^	72.33 (16)	O3^viii^—Ag2—O4^vi^	67.92 (11)
O1^i^—Ag1—O5^iii^	86.46 (10)	O2—Ag2—O4^vi^	125.71 (11)
O1^ii^—Ag1—O5^iii^	119.74 (11)	O2^i^—Ag2—O4^vi^	110.51 (11)
O1^i^—Ag1—O5^iv^	119.74 (11)	O1—Ag2—O4^vi^	177.43 (10)
O1^ii^—Ag1—O5^iv^	86.46 (10)	O1^i^—Ag2—O4^vi^	102.27 (8)
O5^iii^—Ag1—O5^iv^	56.41 (12)	O4^viii^—Ag2—O4^vi^	79.25 (12)
O1^i^—Ag1—O7^v^	65.26 (11)	Ag1^i^—Ag2—Ag1	171.68 (3)
O1^ii^—Ag1—O7^v^	65.26 (11)	O1^i^—Ni1—O7^v^	86.42 (17)
O5^iii^—Ag1—O7^v^	148.99 (7)	O1^i^—Ni1—O6	95.26 (18)
O5^iv^—Ag1—O7^v^	148.99 (7)	O7^v^—Ni1—O6	88.15 (12)
O1^i^—Ag1—O6^v^	107.78 (12)	O1^i^—Ni1—O2	90.92 (15)
O1^ii^—Ag1—O6^v^	107.78 (12)	O7^v^—Ni1—O2	101.25 (13)
O5^iii^—Ag1—O6^v^	132.46 (11)	O6—Ni1—O2	169.08 (15)
O5^iv^—Ag1—O6^v^	132.46 (11)	O1^i^—Ni1—O3	89.93 (14)
O7^v^—Ag1—O6^v^	53.46 (12)	O7^v^—Ni1—O3	170.53 (14)
O1^i^—Ag1—O4^vi^	116.39 (11)	O6—Ni1—O3	100.89 (14)
O1^ii^—Ag1—O4^vi^	164.32 (10)	O2—Ni1—O3	70.05 (11)
O5^iii^—Ag1—O4^vi^	74.98 (10)	O1^i^—Ni1—O4^ix^	175.34 (13)
O5^iv^—Ag1—O4^vi^	98.97 (10)	O7^v^—Ni1—O4^ix^	88.97 (17)
O7^v^—Ag1—O4^vi^	105.40 (12)	O6—Ni1—O4^ix^	83.92 (17)
O6^v^—Ag1—O4^vi^	57.90 (11)	O2—Ni1—O4^ix^	90.62 (14)
O1^i^—Ag1—O4^vii^	164.32 (10)	O3—Ni1—O4^ix^	94.73 (14)
O1^ii^—Ag1—O4^vii^	116.39 (11)	O5^i^—Ni2—O5	178.70 (19)
O5^iii^—Ag1—O4^vii^	98.97 (10)	O5^i^—Ni2—O2^x^	94.43 (14)
O5^iv^—Ag1—O4^vii^	74.98 (10)	O5—Ni2—O2^x^	86.54 (14)
O7^v^—Ag1—O4^vii^	105.40 (12)	O5^i^—Ni2—O2^xi^	86.55 (14)
O6^v^—Ag1—O4^vii^	57.90 (11)	O5—Ni2—O2^xi^	94.42 (14)
O4^vi^—Ag1—O4^vii^	52.10 (14)	O2^x^—Ni2—O2^xi^	83.6 (2)
O3^vi^—Ag2—O3^viii^	100.81 (18)	O5^i^—Ni2—O3^i^	94.10 (14)
O3^vi^—Ag2—O2	177.72 (14)	O5—Ni2—O3^i^	84.97 (15)
O3^viii^—Ag2—O2	76.92 (10)	O2^x^—Ni2—O3^i^	93.29 (11)
O3^vi^—Ag2—O2^i^	76.92 (10)	O2^xi^—Ni2—O3^i^	176.91 (18)
O3^viii^—Ag2—O2^i^	177.72 (14)	O5^i^—Ni2—O3	84.97 (15)
O2—Ag2—O2^i^	105.35 (15)	O5—Ni2—O3	94.10 (14)
O3^vi^—Ag2—O1	125.84 (12)	O2^x^—Ni2—O3	176.91 (18)
O3^viii^—Ag2—O1	114.65 (11)	O2^xi^—Ni2—O3	93.29 (11)
O2—Ag2—O1	55.82 (11)	O3^i^—Ni2—O3	89.8 (2)
O2^i^—Ag2—O1	66.92 (11)	O1—P1—O2	110.0 (2)
O3^vi^—Ag2—O1^i^	114.65 (11)	O1—P1—O4	109.1 (2)
O3^viii^—Ag2—O1^i^	125.84 (12)	O2—P1—O4	113.2 (2)
O2—Ag2—O1^i^	66.92 (11)	O1—P1—O3	115.89 (19)
O2^i^—Ag2—O1^i^	55.82 (11)	O2—P1—O3	100.46 (15)
O1—Ag2—O1^i^	76.28 (13)	O4—P1—O3	108.1 (2)
O3^vi^—Ag2—O4^viii^	67.92 (11)	O5—P2—O5^xii^	109.1 (2)
O3^viii^—Ag2—O4^viii^	52.70 (12)	O5—P2—O6	110.77 (16)
O2—Ag2—O4^viii^	110.51 (11)	O5^xii^—P2—O6	110.77 (16)
O2^i^—Ag2—O4^viii^	125.71 (11)	O5—P2—O7	110.44 (16)
O1—Ag2—O4^viii^	102.27 (8)	O5^xii^—P2—O7	110.44 (16)
O1^i^—Ag2—O4^viii^	177.43 (10)	O6—P2—O7	105.3 (2)
O3^vi^—Ag2—O4^vi^	52.70 (12)	P2—O7—H7	127.4

Symmetry codes: (i) −*x*, −*y*+1, *z*; (ii) *x*+1/2, −*y*+1, *z*; (iii) *x*, −*y*+3/2, *z*−1/2; (iv) −*x*+1/2, −*y*+3/2, *z*−1/2; (v) −*x*+1/2, −*y*+1/2, *z*−1/2; (vi) −*x*, *y*+1/2, *z*−1/2; (vii) *x*+1/2, *y*+1/2, *z*−1/2; (viii) *x*, −*y*+1/2, *z*−1/2; (ix) −*x*, −*y*, *z*; (x) −*x*, *y*+1/2, *z*+1/2; (xi) *x*, −*y*+1/2, *z*+1/2; (xii) −*x*+1/2, *y*, *z*; (xiii) −*x*, *y*−1/2, *z*−1/2; (xiv) −*x*, *y*−1/2, *z*+1/2; (xv) *x*−1/2, *y*−1/2, *z*+1/2; (xvi) −*x*+1/2, −*y*+3/2, *z*+1/2; (xvii) −*x*+1/2, −*y*+1/2, *z*+1/2.

### Hydrogen-bond geometry (Å, °)

**Table:**

*D*—H···*A*	*D*—H	H···*A*	*D*···*A*	*D*—H···*A*
O7—H7···O6^xvii^	0.86	2.06	2.847 (6)	151.

Symmetry codes: (xvii) −*x*+1/2, −*y*+1/2, *z*+1/2.
